# Comparison of the Diagnostic Yield of EUS Needles for Liver Biopsy: Ex Vivo Study

**DOI:** 10.1155/2017/1497831

**Published:** 2017-09-13

**Authors:** Woo Jung Lee, Lance T. Uradomo, Yang Zhang, William Twaddell, Peter Darwin

**Affiliations:** ^1^Temple University, Philadelphia, PA, USA; ^2^University of Maryland, Baltimore, MD, USA

## Abstract

**Background and Aims:**

EUS-guided liver biopsy is an emerging method of liver tissue acquisition which is safe and had been shown to produce excellent histological yield. There is limited data comparing the diagnostic yield of different FNA needles. We aimed to compare the diagnostic performance of four commercially available 19-gauge FNA needles.

**Methods:**

Four FNA needles and one percutaneous needle were used to perform liver biopsies on two human cadaveric livers: Cook Echotip Procore™, Olympus EZ Shot 2™, Boston Scientific Expect Slimline™, Covidien SharkCore™, and an 18-gauge percutaneous needle (TruCore™, Argon Medical Devices). Each needle obtained biopsies by three, six, and nine complete back-and-forth motions of the needle (“throw”) with a fanning technique. The combined lengths of specimen fragments and the total number of complete portal tracts (CPT) were measured by a blinded pathologist. One-way analysis of variance (ANOVA) and Bonferroni correction were used for statistical analysis.

**Results:**

A total of 52 liver biopsies were performed. The Covidien SharkCore needle had significantly greater number of CPT compared to other FNA needles. The number of “throws” did not impact the number of CPT significantly. There was no statistically significant difference in mean total specimen length between each FNA needle type.

**Conclusion:**

The Covidien SharkCore needle produced superior histological specimen by capturing more CPT, possibly due to its unique needle design.

## 1. Introduction

Recent advances in serologic and imaging techniques have improved diagnostic accuracy of liver disease; however, liver biopsy remains a crucial part of the clinician's diagnostic armamentarium. It provides an accurate diagnosis in up to 90% of patients with nondiagnostic serologic and/or imaging evaluations [1, 2]. In addition, liver biopsy plays a central role in assessing disease severity, prognosis, and diagnosis of specific disease such as cryptogenic cirrhosis and the management of liver transplant recipients [3–5].

There have been recent reports of Endoscopic Ultrasound (EUS) guided liver biopsy (EUS-LB) yielding excellent histological specimens [6, 7]. There is a subset of patients in whom EUS-guided liver biopsy would be reasonable, namely, those who are referred for exclusion of biliary obstruction after exhaustive serologic and imaging evaluations. These patients are routinely referred for EUS for examination of biliary system, and it is logical to perform EUS-guided liver biopsy after nondiagnostic biliary evaluation [8]. A recent multicenter study reported that the diagnostic yield of EUS-LB was comparable to percutaneous and transjugular approaches [6]. There is limited data comparing the diagnostic yield of different FNA needles. We therefore performed an ex vivo study comparing the diagnostic performance of four commercially available 19-gauge FNA needles.

## 2. Methods

### 2.1. Liver Biopsy

**Figure 1:**
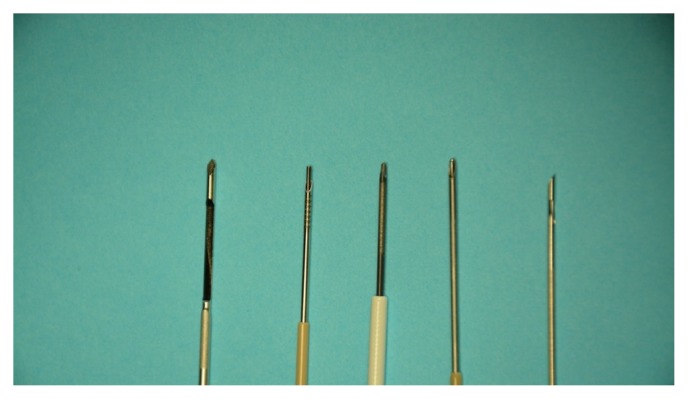
Commercially available FNA needles with different needles tips. From left to right: TruCore percutaneous needles, Olympus EZ Shot 2, Covidien SharkCore, Boston Scientific Expect Slimline, and Cook Echotip Procore.

Two human unembalmed cadaver livers were obtained from the University of Maryland anatomy board. These livers were flushed and procured less than 24 hrs post mortem and did not harbor chronic liver disease. Four commercially available 19-gauge EUS needles were utilized to perform biopsy: Cook Echotip Procore, Olympus EZ Shot 2, Boston Scientific Expect Slimline, and Covidien SharkCore (Figure 1). An 18-gauge percutaneous needle (TruCore, Argon Medical Devices) was used for comparison. Each needle was angled perpendicular to the liver and advanced approximately 6 cm in depth. No stylets were used and full suction was applied for each FNA needle. After initial puncture, a complete back-and-forth motion of the needle was considered one “throw.” Each needle obtained biopsies by three, six, and nine “throws” with a fanning technique. Specimens were collected in separate bottles. All biopsies were taken from within 4-5 mm proximity of each other. Both left and right lobes of each liver were biopsied.

### 2.2. Specimen Processing

**Figure 2:**
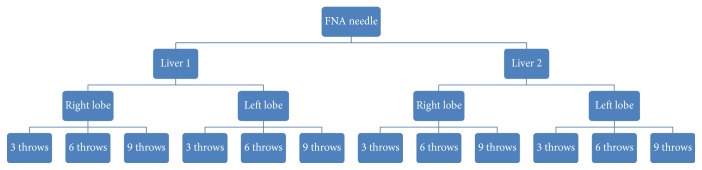
This diagram represents schema of each FNA needles biopsy; different numbers of throws were grouped separately, and biopsies were obtained from right to left lobes of liver from livers 1 and 2, totaling 12 separate specimen jars for each needle. Only 1 specimen was obtained from each lobe of liver for percutaneous reference needle, totaling 4 specimen bottles.

**Figure 3:**
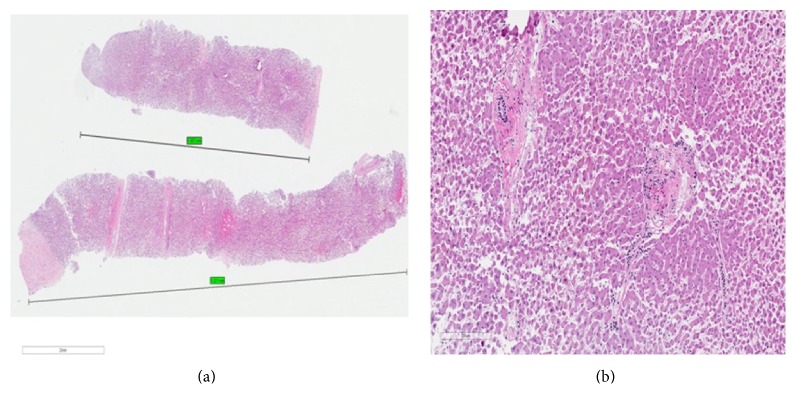
(a) Picture showing intact specimen and its length; (b) picture of specimen showing two complete portal tracts (CPT).

A total of 12 bottles of liver core biopsy specimens were received from each FNA needle and 4 bottles of liver core biopsies were obtained from the percutaneous needle, totaling 52 bottles (Figure 2). They were then fixed in 10% buffered formalin. A pipette was used to transfer the samples from their respective containers into plastic cassettes for processing. The samples were run through the processor, which first dehydrates the tissue using ethanol solution and then clears the alcohol using xylene solution. Wax infiltration was then conducted using paraffin histological wax. The samples were subsequently embedded into blocks using paraffin wax containing plastic. A microtome was used to cut the blocks at 4 microns in thickness, and the cut sections were mounted onto glass slides. Finally, the slides were stained using conventional hematoxylin and eosin staining (Figure 3).

### 2.3. Specimen Analysis

The collected specimens were examined by a blinded pathologist. The diagnostic performance of each needle was determined by measuring the combined length of specimen fragments and total number of complete portal tracts (Figure 3). A complete portal tract (CPT) was defined to contain all three portal structures (portal vein, hepatic artery, and bile duct). The longest single fragment length for each needle was also recorded.

### 2.4. Statistical Analysis

One-way analysis of variance (ANOVA) was used to analyze a significant difference in mean number of portal triads and specimen length utilizing STATA software, College Station, TX.

The Bonferroni correction allowed for identification of the specific difference between each needle type. Analysis was conducted for the three, six, and nine “throw” groups in each needle type. The “throw” groups were also combined.

## 3. Results

**Figure 4:**
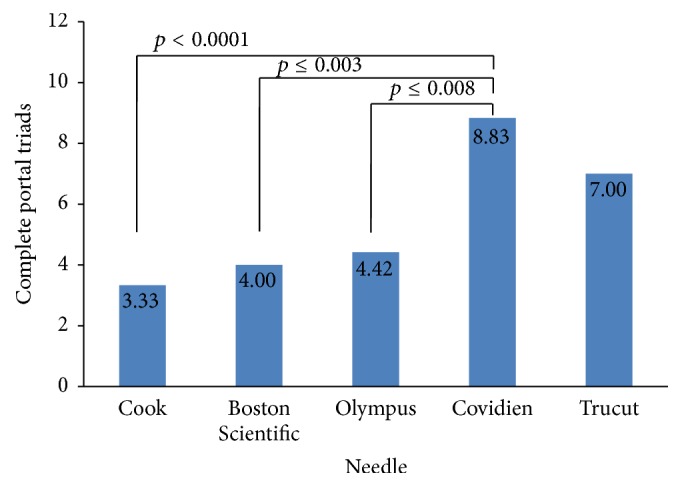
Comparison of CPT obtained from each FNA needle using one-way ANOVA analysis with Bonferroni correction.

There was a significantly greater number of CPT with the Covidien needle compared to other FNA needles in the one-way ANOVA analysis with Bonferroni correction (Figure 4). The mean CPT for Covidien needle was 8.83. This was significantly greater than the mean CPT for the Cook, Boston Scientific, and Olympus needles which were 3.33 (*p* < 0.001), 4 (*p* ≤ 0.003), and 4.42 (*p* ≤ 0.008), respectively. The mean CPT for the Trucut percutaneous needle was 7 (*p* > 0.05).

The analysis stratified by number of “throws” showed a significant difference in CPT yield only in the groups where 3 “throws” were performed (Table 1). In this group, the mean CPT for the Covidien needle was 9.5, whereas the Cook, Boston Scientific, and Olympus needles were 2.75 (*p* ≤ 0.01), 3 (*p* ≤ 0.013), and 1.5 (*p* ≤ 0.003), respectively. Overall, the number of “throws” did not have a significant independent impact on CPT (*p* ≤ 0.17). The two-way ANOVA model which included both needle type and number of “throws” confirmed higher CPT (*p* ≤ 0.0002) with the Covidien needle.

There was no statistically significant difference in mean total specimen length between each FNA needle type (*p* ≤ 0.11, Table 2). While the Cook needle had the lowest mean total specimen length (2.98 cm) and the Covidien needle had the highest mean total specimen length (5.07 cm), there was no difference in mean specimen length between the three (3.23 cm), six (4.76 cm), and nine (4.79 cm) “throws” groups (Table 3). Stratification by number of “throws” also showed no difference in mean specimen length between each needle type (three “throws” *p* ≤ 0.55; six “throws” *p* ≤ 0.62; nine “throws” *p* ≤ 0.44). Among the FNA needles, the single longest fragment was longest in the Covidien group (0.90 cm), but there was not a significant difference found between groups (*p* ≤ 0.31). The Trucut needle, not surprisingly, showed the longest fragment at 1.38 cm.

## 4. Discussion

Obtaining an adequate sample from liver biopsy for accurate interpretation and diagnosis of underlying disease depends on multiple factors. One of the most important factors is specimen size, as it should be large enough to view a representative amount of parenchyma. The optimal specimen length has been studied previously, and there lacks a uniformly recommended length to make a histological diagnosis [1, 9]. Some authors argue minimal specimen of 2 cm for chronic liver disease such as hepatitis B or C, as biopsy specimen less than 2 cm had reduced accuracy in grading and staging [10]. For cirrhotic patients, biopsy specimen with short length failed to recognize cirrhosis in up to 20% of cases [11]. Other authors accept specimen length of 1.5 cm to be adequate for assessing many other liver diseases [1]. The number of complete portal tracts is another important parameter in assessing the quality of biopsy specimen. While some authors have proposed the adequate number of portal tracts to be greater than 11 [10], studies have shown most biopsy specimens (percutaneous or transjugular approach) underperform with mean CPT of 6.8–7.7 [12]. A review of transjugular biopsy samples yielded mean CPT of 6.5 and mean aggregate specimen length of 12 mm [13]. A liver biopsy specimen with 6–8 CPT may be considered adequate [1].

Using specimen length and number of CPT, our study compared the performance of each EUS needle in obtaining liver biopsy. A good correlation between specimen length and CPT was observed, as the needle with lowest specimen length had the least CPT and the needle with highest specimen length resulted in the highest CPT. Although the differences among specimen lengths from each needle were not statistically significant, this may be attributable to the relatively small sample size. The Covidien SharkCore needle yielded the highest number of CPT, and this difference was statistically significant. The discrepancy of greater number of CPT but not specimen lengths from the SharkCore can be explained by the unique design of the SharkCore needle. The needle tip is designed with six cutting edge surfaces and an opposing bevel to catch tissue as it is sheared off, which allows acquisition of cohesive units of tissue with intact architecture. A recent study by Schulman et al. [14] demonstrated the significantly greater number of CPT obtained using the novel 19-gauge needle (Covidien SharkCore) compared to other needles (Cook Procore™, Boston Scientific Expect™, and 18-gauge percutaneous needles), further supporting our data.

Another interesting finding from our study is the weak correlation between the number of to-and-fro needle movements (“throws”) and the quality of biopsy specimens. More needle movements did not result in higher specimen length or the CPT. The CPT of all needle specimens for 9 throws was greater when compared to 3 throws, but this was not statistically significant. This finding is clinically relevant as it suggests less needle movements may provide specimen of comparable quality to higher number of movements while causing less trauma to the target organ.

The main limitation from our study was its ex vivo design. There are a multitude of factors that influence the performance of a needle in an actual clinical setting, such as patient factors (anatomy, comorbidities which may affect stability and duration of the procedure, etc.) and operator factors (operator experience, amount and depth of each pass he/she feels comfortable performing, etc.), and these factors were obviously not included in this study. Also the true endpoint in evaluation of biopsy needle, which is the histologic diagnosis of underlying liver disease, could not be assessed in this study due to its ex vivo design. Another important limitation is the small sample size. A total of twelve biopsies were performed from two livers for each needle, and only one needle from each commercial vendors were used. The higher specimen length from the SharkCore needle and the higher CPT from 9 throws may have been statistically insignificant due to this sample size issue and need to be further elucidated. However, despite the limitations of this study, our data does provide preliminary evidence to suggest there are differences in performance of each needle.

In conclusion, our study examined head-to-head comparisons in performance of four different EUS needles in liver biopsy. Our data showed objective evidence that suggests the SharkCore needle may perform better than other needles in obtaining liver biopsy. Given the ex vivo design and small sample size of this study, a follow-up trial should be performed to confirm our finding.

## Figures and Tables

**Table 1:** Mean complete portal tracts by needle type and number of throws.

	Number of throws		
	Three	Six	Nine
Cook	2.75	3.75	3.5
Boston Scientific	3	4	5
Olympus	1.5	5.5	6.25
Covidien	9.5	5.75	11.25
	*p* ≤ 0.0018	*p* ≤ 0.28	*p* ≤ 0.053

**Table 2:** Total specimen length by needle type.

	Total specimen length (cm)
Cook	4.73
Boston Scientific	2.98
Olympus	4.26
Covidien	5.07
Trucut	1.55

**Table 3:** Total specimen length by needle type and number of throws.

	Number of throws		
	Three	Six	Nine
Cook	2.25	3.43	3.25
Boston Scientific	3.82	5	5.35
Olympus	2.55	4.97	5.25
Covidien	4.28	5.63	5.33
	*p* ≤ 0.55	*p* ≤ 0.62	*p* ≤ 0.44
